# Viscosity-enhanced droplet motion in sealed superhydrophobic capillaries

**DOI:** 10.1126/sciadv.aba5197

**Published:** 2020-10-16

**Authors:** Maja Vuckovac, Matilda Backholm, Jaakko V. I. Timonen, Robin H. A. Ras

**Affiliations:** 1 Department of Applied Physics, Aalto University, P.O. Box 15100, 02150 Espoo, Finland.; 2 Department of Bioproducts and Biosystems, Aalto University, P.O. Box 16000, 02150 Espoo, Finland.

## Abstract

It is well known that an increased viscosity slows down fluid dynamics. Here we show that this intuitive rule is not general and can fail for liquids flowing in confined liquid-repellent systems. A gravity-driven, highly viscous glycerol droplet inside a sealed superhydrophobic capillary is moving more than 10 times faster than a water droplet with three-orders-of-magnitude lower viscosity. Using tracer particles, we show that the low-viscosity droplets are rapidly rotating internally, with flow velocities greatly exceeding the center-of-mass velocity. This is in stark contrast to the faster moving high-viscosity droplets with nearly vanishing internal flows. The anomalous viscosity-enhanced flow is caused by a viscosity-suppressed deformation of the droplet-air interface and a hydro- and aerodynamic coupling between the droplet and the air trapped within the micro/nanostructures (plastron). Our work demonstrates the unexpected role of the plastron in controlling fluid flow beyond the mere reduction in contact area and friction.

## Teaser

The general rule that viscosity slows droplet motion fails for a droplet in a superhydrophobic tube closed in one or both ends.

## INTRODUCTION

Viscosity η is a measure of resistance of fluid to flow under an external force or pressure gradient Δ*P* (*1*). Viscosity plays an important role, as it limits fluid flow and mass transfer at all length scales and in a wide range of systems from industrial processes to biological circulatory networks (*2*). Often, it is desirable to have the fluid flow fast and effortlessly. In a simple model, volumetric flow rate *Q* inside a pipe, whether a blood vessel or a microfluidic device, scales as *Q* ∝ Δ*P*/η in the laminar regime (*1*). The reduced flow rate due to increased viscosity can be counteracted by increasing the driving pressure gradient, but only to a limited extent, as it will also increase the mechanical stress on the pipe. Thus, finding approaches for making viscous liquids flow faster without the need to increase the driving force is of considerable technological interest.

One extensively studied approach is to use superhydrophobic coatings (*3*). Superhydrophobic coatings can support a metastable air layer, a plastron, between the solid wall and liquid phase (Cassie-Baxter state) (*4*–*8*). The presence of a plastron reduces the solid-liquid contact area and leads to high apparent contact angles (*5*, *9*, *10*) and small frictional forces (*11*, *12*). Liquids moving on top of the plastron can show macroscopic viscous slip that leads to nonzero flow at the apparent solid-liquid boundary (*13*–*15*), which has been shown to enhance the liquid flow up to 50 to 65% (*16*). However, increasing fluid viscosity still leads to a reduction in the flow rate.

### Experimental observation of viscosity-enhanced fluid flow

**Fig. 1:**
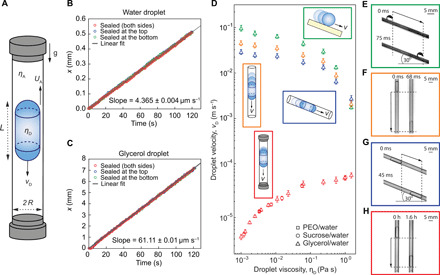
Experimental observation of viscosity-enhanced droplet motion inside a sealed superhydrophobic capillary. (**A**) Schematic depiction (not to scale) of the droplet (viscosity η_D_ and length *L*) moving in a superhydrophobic capillary with radius *R*. The air (viscosity η_A_) flows from the bottom (high-pressure side) to the top (low-pressure side) of the capillary through the plastron with an average velocity *U*_A_, resulting in a downward motion of the droplet with a center-of-mass velocity *v*_D_. (**B**) Water droplet and (**C**) glycerol droplet positions as a function of time inside the sealed superhydrophobic capillary and superhydrophobic capillaries with one closed end on the top or the bottom. (**D**) Droplet velocity as a function of fluid viscosity in different geometries with superhydrophobic boundaries: (**E**) droplet on an inclined flat superhydrophobic surface, (**F**) droplet inside an open vertical superhydrophobic capillary, (**G**) droplet inside an open inclined superhydrophobic capillary, and (**H**) droplet inside a sealed vertical superhydrophobic capillary. Viscosity was varied by using solutions of polyethylene oxide (PEO) in water (▫) with volume fraction of PEO ranging from 0.1 to 20%, solutions of sucrose in water (○) with volume fraction of sucrose ranging from 40 to 72%, and mixtures of glycerol and water (▵) with volume fraction of glycerol ranging from 0 to 100% (see table S1 for details). Each data point in sealed superhydrophobic tube measurements is an average of 10 measurements for 10 capillaries. Error bars denote SDs. Velocities reported for sealed superhydrophobic tubes and for high-viscosity droplets in open superhydrophobic geometries are terminal velocities. Other reported velocities (for low-viscosity liquids on other superhydrophobic geometries) were measured from droplets that were still in accelerating motion.

**Fig. 2:**
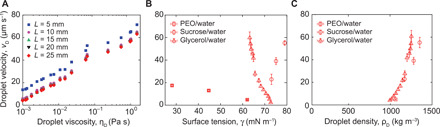
Center-of-mass velocity of droplets moving inside sealed superhydrophobic capillaries. *v*_D_ (**A**) Center-of-mass droplet velocity as a function of viscosity η_D_ for different droplet lengths *L*. (**B**) Center-of-mass droplet velocity as a function of surface tension γ (*L* = 15 mm). (**C**) Center-of-mass droplet velocity as a function of density ρ_D_ (*L* = 15 mm). Each data point is an average of five measurements, and the error bars denote SDs.

In this work, we show that the applicability of superhydrophobic coatings goes beyond the slip-induced flow enhancement. Using vertically oriented superhydrophobic capillaries closed from one or both ends as a model system (Fig. 1, A to C), we demonstrate that the superhydrophobic coating can, counterintuitively, make viscous fluids flow faster than less viscous fluids when driven by their own weight in gravitational field (Fig. 1 and figs. S1 and S2). We demonstrate this using a commercially available superhydrophobic coating solution (Hydrobead) that we apply to prepare semitransparent superhydrophobic coatings on the inner surfaces of glass capillaries with an inner radius of *R* = 2.10 ± 0.05 mm (see Materials and Methods and figs. S1 and S2). Droplets of various liquids like water and glycerol adopt a cylindrical form with hemispherical ends (total length *L*) when placed inside the tubes due to the confinement and surface tension (Fig. 1A). The droplets driven by gravity flow downward in the tubes with the center-of-mass droplet velocity *v*_D_ constant over time. However, the velocity increases with the viscosity of the droplet η_D_ (Figs. 1, D and H, and 2B). This applies for all studied viscosities ranging from ca. 1 to more than 1000 mPa s. For example, velocity of a highly viscous droplet of glycerol (~1000 mPa s) can be an order of magnitude larger than velocity of a similarly sized water droplet with three-orders-of-magnitude smaller viscosity (~1 mPa s) (Fig. 2B). This core observation is in stark disagreement with the conventional droplet motion on inclined planar superhydrophobic surfaces (Fig. 1, D and E) (*17*, *18*), where the droplet velocity is inversely proportional to the droplet viscosity. In addition, droplets moving inside vertical (Fig. 1, D and F) and tilted (Fig. 1, D and G) superhydrophobic capillaries with open ends show the conventional expected behavior, i.e., decrease in velocity with increasing viscosity of the fluid (Fig. 1D). In the remaining of article, we present a thorough experimental and theoretical study of this seemingly anomalous viscosity-enhanced motion and show how it arises from an unexpected combination of coupling of hydro- and aerodynamic flows (inside the droplet and in the plastron) and viscosity-suppressed penetration of the droplet into the micro/nanostructures of the superhydrophobic coating.

## RESULTS AND DISCUSSION

### Failure of existing theoretical models to explain viscosity-enhanced flow

First, the known concept of slip-induced drag reduction (*15*, *19*), ubiquitous for contact line–free, continuous fluid flow on superhydrophobic coatings, predicts an inverse proportionality between the friction factor (*19*) and the Reynolds number (Re = 2*R*ρ*U*/η_D_, where ρ is the fluid density and *U* is the average flow velocity) in a superhydrophobic pipe (*20*). This translates to an increase in the friction factor with increasing viscosity and would predict slower fluid dynamics with increased viscosity, in contrast to what we observe experimentally.

Second, the superhydrophobic surface-induced drag reduction in our single-droplet system will mainly come from the contact angle hysteresis of the surface (*21*), which is independent of the fluid viscosity for slow, quasi-equilibrium droplet motion, and cannot explain our experimental observation.

Third, the Landau-Levich-Bretherton (LLB) theory (*22*–*27*), which can describe a fluid drop moving through a capillary filled with another fluid and where a lubricating liquid film is created between the drop and the capillary wall through viscous entrainment of the outer fluid, is not relevant in our system. In our case, the LLB theory would predict the thickness of the lubricating film to scale as δ_LLB_ ∼ *R*Ca^2/3^, where Ca = *v*_D_η_A_/γ is the capillary number, η_A_ ≈ 18.7 μPa s is the viscosity of the surrounding air, and γ is the surface tension of the droplet. An estimate of this film thickness for water (γ = 0.0727 N/m) and glycerol (γ = 0.0632 N/m) droplets gives δ ≈ 2 and 14 nm, respectively, which is much thinner than the plastron created within the microstructured capillary wall (roughness δ = 7.2 ± 1.2 μm; see fig. S2C).

### Experimental observations on the droplet velocity

First, control experiments performed with tubes without a superhydrophobic plastron (capillaries with the inner surface coated with a smooth hydrophobic coating or a superhydrophobic coating in the Wenzel state) (fig. S3) (*8*, *28*) show no droplet motion at all. This proves that the existence of the plastron is critical for the observed phenomenon. Second, droplet velocity is not strongly depending on the droplet size (Fig. 2A), suggesting that forces driving and resisting droplet flow scale similarly with droplet length *L*. Third, the droplet velocity depends on the surface tension; however, the correlation between droplet velocity and surface tension (data do not fall on a single master curve) suggests that surface tension is not a key parameter (Fig. 2B). Fourth, droplet velocity increases with the density of the droplet (Fig. 2C), but one needs to notice that in our system with increasing the density, the viscosity increases as well.

Together, these observations point toward a physical picture where there is a coupling between the droplet motion and the air flow in the plastron: As the droplet moves downward, it acts as a piston compressing air below the droplet (Fig. 1A). This leads to a pressure gradient in the air between the high-pressure side (below the droplet) and the low-pressure side (above the droplet). The pressure difference drives the air through the plastron from below to above of the droplet, allowing the droplet to slowly move down through the capillary.

### Viscosity-suppressed flows inside moving droplets

**Fig. 3:**
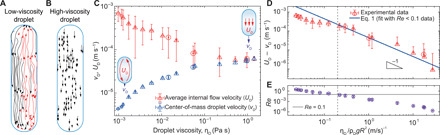
Fluid flows inside droplets. (**A**) The flow profile inside a low-viscosity (30% glycerol/water, η_D_ = 2.5 mPa s) and (**B**) high-viscosity (η_D_ = 1499 mPa s) droplet. Images show experimentally obtained trajectories of particles traced for 60 s. (**C**) Experimentally obtained center-of-mass droplet velocity, *v*_D_ (blue symbols), and average internal flow velocity, *U*_D_ (red symbols), as a function of viscosity for all systems [left to right: PEO/water solutions (▫) with volume fraction of PEO 0.1%, sucrose/water solutions (○) with volume fraction of sucrose from 40 to 72%, and glycerol/water solutions (Δ) with volume fraction of glycerol from 0 to100%]. Each data point is the average of five measurements, while the error bars denote SDs. (**D**) Internal droplet flow as a function of η_D_/ρ_D_*gR*^2^. The solid blue line is a fit of Eq. 1 with *Re* < 0.1 (laminar) data. For low droplet viscosities, inertial [*F*_D, ρ_ ~ ρ_D_*RL*(*U*_D_ − *v*_D_)^2^] and viscous [*F*_D, η_ ~ η_D_*L*(*U*_D_ − *v*_D_] dissipative forces are of similar importance (fig. S6), and a purely viscous model cannot capture the flow in this regime. We have excluded the 9 and 20% PEO/water solutions from (C), since these have concentrations exceeding the overlap concentration (*32*), c*, resulting in entangled polymers and non-Newtonian flow (see the Supplementary Materials for calculations). (**E**) Reynolds number (*Re* = 2*R*ρ_D_(*U*_D_ − *v*_D_)/η_D_) as a function of η_D_/ρ_D_*gR*^2^. A transition from droplets with purely viscous flow (*Re* < 0.1 ≪ 1 for high viscosities) to droplets with viscous and inertial forces of similar importance (*Re* ≈ 1 for low viscosities) is highlighted. The error bars are error propagations including the SDs of *v*_D_, *R*, ρ, and η_D_ as well as the average absolute SD of *U*_D_. In all plots, droplet size is fixed, *L* = 15 mm.

The next critical piece of the puzzle toward understanding why higher-viscosity droplets move faster in the capillary comes from the internal flows inside the droplets. We used tracer particles to visualize the flows inside the droplets moving down the capillaries (figs. S4 and S5 and movies S1 to S3). The tracer particle experiments show a consistent asymmetric, “rolling” flow inside low- and intermediate-viscosity (η_D_ < 62 mPa s) droplets (Fig. 3A). In contrast, a symmetric, “sliding” plug flow is seen inside the high-viscosity (η_D_ > 62 mPa s) droplets (Fig. 3B). The transition from axisymmetric core-annular flow at high viscosities to the rolling flow for low and intermediate viscosities is supported by previous studies on exchange flow in vertical pipes, finding that steady, bidirectional, axisymmetric core-annular flow in a cylindrical liquid-liquid geometry only occurs at high-viscosity ratios between the two fluids (η_D_/η_A_ > 75 to 300) (*29*–*31*). Because the viscosity ratio for our lowest-viscosity system (pure water) is η_D_/η_A_ = 53, it is reasonable that we do not have axisymmetric core-annular flow here.

We quantify the internal flows by defining the average internal flow velocity (*U*_D_) in the reference frame of the tube in cylindrical geometry as *U*_D_ = ∫ 2π*urdr*/ ∫ 2π*rdr*, where *u* = *u*(*r*) is the speed at distance *r* from the tube axis. This is approximated as a summation over the tracer particles that are approximately evenly distributed in the narrow two-dimensional focal plane (Fig. 3, A and B), as $U_{D}\approx \Sigma_{i=1}^{n}r_{i}u_{i}/\Sigma_{i=1}^{n}r_{i}$, where *n* is the number of tracer particles*, u_i_* is the particle velocity and *r_i_* is the particle distance from the tube axis (see Materials and Methods and the Supplementary Materials for details). Unexpectedly, the average internal flow velocity decreases with droplet viscosity, whereas the center-of-mass velocity increases with it (Fig. 3C). Furthermore, the average internal flow velocities inside the slowly moving, low-viscosity droplets are considerably higher than the center-of-mass velocities (*U*_D_ ≈ 0.7 mm s^−1^ versus *v*_D_ = 0.004 mm s^−1^ for low-viscosity droplets). Thus, the low-viscosity droplets that move very slowly inside the capillaries (almost immobile at short time scales) are actually going through vigorous internal mixing that is only visible when using tracer particles (movie S1). On the other hand, the velocity of the tracer particles in the high-viscosity droplets like glycerol approaches the center-of-mass droplet velocity in the capillary, which is *U*_D_ ≈ *v*_D_ ≈ 0.06 mm s^−1^ (movie S3).

Theoretically, the fluid flow inside the droplets is determined by a balance between viscous dissipation *F*_D, η_~η_D_*L*(*U*_D_ − *v*_D_) and the driving force *F* ∼ ρ_D_*gLR*^2^, yielding

$$U_{D}-v_{D}∼\frac{\rho_{D}gR^{2}}{\eta_{D}}$$ (1)

The scaling law of Eq. 1 is verified experimentally using data from tracer particle experiments (Fig. 3D) for cases where the flow is laminar (Reynolds number Re ∼ *R*ρ_D_(*U*_D_ − *v*_D_)/η_D_ < 0.1 ≪ 1; Fig. 3E). Thus, increasing the viscosity of the fluid suppresses fluid dynamics inside the droplets, as expected.

### Coupling of hydro- and aerodynamic flows to derive scaling law for droplet velocity

As a result of the droplet acting as a piston pressurizing the air below the droplet, we model the air inside the plastron (with average thickness δ ≪ *R*) to move upward at an average velocity of *U*_A_. We assume viscous flow in both the liquid and the air and that the air remains uncompressed. The volumetric flow rates of the droplet, *Q*_D_~*v*_D_*R*^2^, and the air, $Q_{A}∼\int_{R-\delta }^{R}rU_{A}dr=\frac{1}{2}\;U_{A}(2R\delta -\delta^{2})∼U_{A}R\delta$ (since δ ≪ *R*), must be in balance (*Q*_D_ = *Q*_A_), giving the average air velocity as *U*_A_ ∼ *v*_D_*R*/δ. Dissipation in the air generally occurs through viscous (*F*_A, η_ ∼ η_A_*RLU*_A_/δ ∼ η_A_*Lv*_D_*R*^2^/δ^2^) and inertial ($F_{A,\rho }∼\rho_{A}\mathrm{RL}U_{A}^{2}∼\rho_{A}R^{3}Lv_{D}^{2}/\delta^{2}$) friction forces. In our system, the viscous dissipation in the air film (Re_air_ ≪ 0.01 in all cases) always dominates (fig. S6) and is balanced by the pressure difference Δ*P* in the gas phase between the two sides of the droplet. We measured the pressure difference using a manometer and found out that it scales as Δ*P* ~ ρ_D_*gL* (fig. S1B), so that the driving force for the air flow is *F*_ρ_ ∼ Δ*PR*δ ∼ ρ_D_*gLR*δ (where the cross-sectional area of the air film scales as *R*δ since δ ≪ *R*). Balancing friction and driving force yield ρ_D_*gLR*δ ∼ η_A_*Lv*_D_*R*^2^/δ^2^ and thus

$$v_{D}∼\frac{\rho_{D}g\delta^{3}}{\eta_{A}R}$$ (2)

From Eq. 2, the center-of-mass velocity of the droplet appears not to depend on droplet size, which is in excellent agreement with experimental findings (Fig. 2A and fig. S7) for long droplets. Equation 2 also predicts a strong increase in the velocity of the droplet on increasing the plastron thickness, which is intuitively clear since this allows for higher volumetric flow rate of air upward in the plastron, making the droplet move faster downward. However, Eq. 2 also predicts that the relation between velocity and density can be described by a linear function going through origin, which is not experimentally observed (Fig. 2C). Because all other variables on the right-hand side are fixed, Eq. 2 can describe our system only if the plastron thickness δ is not a constant.

### Introducing viscosity-dependent plastron thickness

**Fig. 4:**
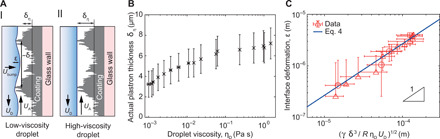
Deformation of the droplet interface. (**A**) Schematic depictions of the interface deformation. (I) Low-viscosity droplet where δ_η_ is the actual plastron thickness, ε is the deformation of the droplet interface into the plastron, and δ is a roughness unit swept by droplet during time τ. (II) High-viscosity droplet that remains smooth. (**B**) The actual plastron thickness δ_η_ as a function of viscosity η_D_. (**C**) Interface deformation as a function of (γδ^3^/*R*η_D_*U*_D_)^1/2^. The data (red) were calculated using Eq. 3 and follows the scaling law predicted by Eq. 4 (solid blue line). The error bars are error propagations including the SDs of *v*_D_, *R*, ρ, δ, and η_D_, as well as the average absolute SD of *U*_D_. In (C), the data from the glycerol drop is not included, since its interface is assumed to be flat (ε_glycerol_ = 0).

To properly describe our experimental data with Eq. 2, we introduce a varying plastron thickness that depends on viscosity: δ_η_(η_D_, ρ_D_, γ) ≡ δ − ε(η_D_, ρ_D_, γ), where δ_η_ is the actual average air film thickness around the moving droplet and ε represents a deformation of the fluid interface compared to a case of a flat liquid-gas interface (Fig. 4A). Equation 2 is rewritten as $g/\eta_{A}R∼v_{D}/\rho_{D}\delta_{\eta }^{3}=c_{g}$, where *c*_g_ is a constant (since the left-hand side *g*/η_A_*R* is constant in our experiments). By assuming a nondeformed, flat interface for highly viscous droplets ε(η_D_ → ∞ ) = 0*, c*_g_ is calculated as

$$\frac{v_{D}}{\rho_{D}{\left(\delta -\varepsilon \right)}^{3}}=c_{g}\equiv {\left(\frac{v_{D}}{\rho_{D}\delta^{3}}\right)}_{\text{glycerol}}=1.3\pm 0.7\cdot 10^{8}\frac{m}{s\;\text{kg}}$$ (3)

Thus, a glycerol droplet is assumed to be viscous enough to have a (nearly) smooth interface (ε_glycerol_ = 0), and we, hence, use the glycerol case as a fixed system to relate all other droplets to. Using the experimental values for *v*_D_, ρ_D_, and δ as input parameters in Eq. 3, the interface deformation for all other droplets can be numerically determined as ε = δ − (*v*_D_/*c*_g_ρ_D_)^1/3^ (Fig. 4B). We find the actual plastron thickness to decrease from δ_η_ = 7.2 ± 1.5 μm at high viscosities to δ_η_ = 3.2 ± 1.3 μm at low viscosities (Fig. 4B). Using the actual plastron thickness, we recalculate the dissipative forces and confirm that the viscous dissipation in the air film still is the dominating factor (fig. S6).

### Mechanism for viscosity-dependent plastron

The mechanism for reduction in the plastron thickness for low-viscosity fluids is proposed in the following. The droplet-plastron interface tends to curve into the superhydrophobic coating because of the Laplace pressure. This can be considered as the growth of fluid “bumps” into the microstructures. The bump growth follows Stokes equation ∇*p* ∼ η_D_∇^2^*U*_bump_ ∼ η_D_*U*_bump_/δ^2^, where ∇*p* is the pressure gradient, *U*_bump_ is the lateral (radial) growth velocity of the bump toward the wall, and δ is the roughness length scale of the superhydrophobic coating (Fig. 4A). In our system, the pressure *p* ∼ γ/*R* is the internal Laplace pressure of the drop, and the pressure gradient over a bump of size ε is ∇*p* ∼ γ/*R*ε. This gives a scaling for the growth velocity of the interfacial bump as *U*_bump_ ∼ γδ^2^/*R*εη_D_. The interface has time to deform into the roughness of the wall until the fluid in the droplet has flowed past that position of the wall (i.e., one “roughness unit” δ; Fig. 4A). This time scales as τ ∼ δ/*U*_D_, and the interface, thus, has the time to grow a distance ε ∼ *U*_bump_τ ∼ γδ^3^/*R*εη_D_*U*_D_, giving

$$\varepsilon ∼{\left(\frac{\gamma \delta^{3}}{R\eta_{D}U_{D}}\right)}^{1/2}$$ (4)

which will be valid if the liquid does not completely fill the coating (ε < δ). We find excellent agreement between the interface deformation estimates achieved from Eq. 3 and the theoretical model of Eq. 4 for all droplets (Fig. 4C).

### Direct observation of deformation of the droplet-plastron interface

**Fig. 5:**
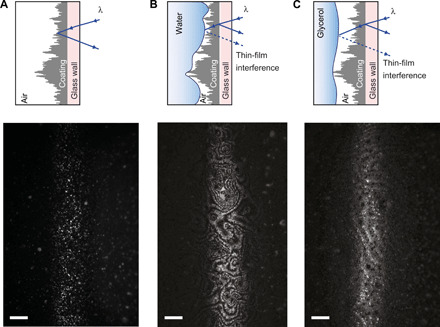
Visualization of the interface between the moving liquid droplet and the plastron using confocal RICM. (**A**) Scheme of reflection and scattering of light from an empty capillary with a superhydrophobic coating (top) and the corresponding RICM image (below). The capillary tube is positioned vertically in the RICM image, and only ca. 150-μm-wide section is in focus due to curvature of the capillary tube. White dots in RICM image indicate locations in the superhydrophobic coating (with above average roughness) that scatter light strongly. (**B**) Scheme of the thin-film interference effect for low-viscosity liquid (water droplet, η_D_ = 0.001 Pa s) and a corresponding RICM image. (**C**) Scheme of the thin-film interference effect for high-viscosity liquid (glycerol droplet, η_D_ = 1 Pa s) and a corresponding RICM image. (A to C) In all RICM images, wavelength is 458 nm, and scale bars correspond to 100 μm.

To directly verify the interface deformation for low-viscosity droplets, we imaged the liquid-gas interface of droplets moving inside the capillaries using confocal reflection interference contrast microscopy (RICM) (Fig. 5 and movies S4 and S5). Our setup was similar to one previously used to study lubricant dynamics of lubricated surfaces (*33*), with the exception that the optical beam path and objective lens were rotated 90° to horizontal orientation to allow imaging of vertically oriented capillaries. When the plastron is not present, the light is scattered by the micro/nanostructured coating and reflected from the coating/capillary interface, and thus, there are no interference fringes formed (Fig. 5A). In the presence of a droplet and a plastron, light reflected from the air/liquid and the capillary/coating interfaces interfere with one another, leading to a fringe pattern (Fig. 5, B and C). In the case of the water droplet, the RICM image shows an interference pattern with densely packed interference contours, indicating a plastron with a spatially varying thickness (Fig. 5B). On the other hand, for a glycerol droplet, the RICM image shows sparsely packed interference contours, indicating the presence of a plastron with an essentially constant thickness (Fig. 5C). From these direct observations from moving droplets, we conclude that the droplet-plastron interface is considerably deformed for low-viscosity fluids, resulting in thinner plastron and, eventually, to the counterintuitive slowing down of low-viscosity fluids.

### Conclusions and outlook

We have shown that the seemingly anomalous viscosity-enhanced flow of droplets in closed superhydrophobic capillaries arises from intimate coupling of fluid flows inside the droplet and in the plastron. Specifically, it arises from a nontrivial but notable reduction in the average plastron thickness for low-viscosity droplets compared with high-viscosity ones: Thinner plastron for low-viscosity droplets reduced the volumetric flow rate of air moving upward, leading to a reduction in the center-of-mass velocity of the droplet going downward. Reduction in the average plastron thickness itself results from radial (lateral) flow of the droplet into the plastron that is favored for low-viscosity droplets due to their lower viscosity.

We have also verified our findings using capillaries coated with another superhydrophobic coating with smaller roughness (fig. S8, A to C). Therein, similar viscosity-enhanced droplet motion was observed (fig. S8, D and E). These two experimental verifications and our theoretical considerations suggest that the viscosity-enhanced droplet motion is a general phenomenon for closed superhydrophobic capillaries. Thus, we conclude that coupling of the hydrodynamics in the droplet and aerodynamics in the plastron is important in superhydrophobic capillaries and can lead to counterintuitive phenomena such as viscosity-enhanced droplet motion, with potential major applications for liquid transport especially in contexts of micro- and nanofluidics.

## MATERIALS AND METHODS

### Superhydrophobic capillaries coated with Hydrobead

Nuclear magnetic resonance (NMR) sample capillaries (Wilmad-LabGlass SP Scienceware) with 4.20-mm inner diameter and 18-cm length were cut from one side to obtain open-ended capillaries (length after cutting, ~15 cm). Capillaries were cleaned by ultrasonication in an alkaline solvent (Deconex 11 Universal, VWR) for 30 min, rinsed thoroughly with Milli-Q water, and dried under nitrogen flow. Afterward, capillaries were coated with Hydrobead Standard (a commercially available superhydrophobic coating) by placing one drop of solution into a capillary, taking care that the pipette does not touch the inside wall of the capillary. Capillaries were dried carefully by nitrogen flow followed by 10-min annealing at 110°C. The resulting capillaries are superhydrophobic and semitransparent.

### Superhydrophobic capillaries coated with Glaco

The NMR sample capillaries and cleaning procedure are the same as described for superhydrophobic capillaries coated with Hydrobead. Capillaries were coated with Glaco Mirror Zero Coat (a commercially available superhydrophobic coating) by filling the capillary with the solution using a pipette. Afterward, capillaries were dried carefully at ambient conditions and annealed for 10 min at 110°C. The resulting capillaries are superhydrophobic and transparent.

### Hydrophobic capillary

The NMR sample capillaries and cleaning procedure are the same as described for superhydrophobic capillaries coated with Hydrobead. Capillaries were hydrophobized by immersion in 0.002 M octadecylphosphonic acid/tetrahydrofuran solution (ODPA/THF) for 35 min. Afterward, tubes were annealed in an oven for 48 hours at 120°C. The resulting capillary is hydrophobic and transparent (fig. S3A). The ODPA/THF solution was prepared by dissolving ODPA powder (Sigma-Aldrich) into THF (Sigma-Aldrich) by ultrasonication for completely dissolving the ODPA powder.

### Superhydrophobic capillary in the Wenzel state

The Wenzel state was obtained using superhydrophobic capillaries coated with Hydrobead and a droplet of 50% water/ethanol solution. The droplet was in the Wenzel state, as can be seen from the wetting of the tube along the contact area (fig. S3B).

### Synthesis of planar superhydrophobic surfaces

Microscope glass slides were cleaned by ultrasonication in an alkaline solvent (Deconex 11 Universal, VWR), rinsed thoroughly with Milli-Q water, and dried under nitrogen flow. Afterward, Hydrobead Standard was applied on glass slides and dried for 10 min.

### Contact angle measurements

Contact angles were measured with the sessile drop method by using a conventional optical tensiometer (Attension Theta) with an automated liquid pumping system. Advancing contact angles were measured by placing a 0.2-μl droplet on the surface and increasing its volume to 20 μl, at a rate of 0.05 μl/s. Receding contact angles were measured by decreasing droplet volume at a rate of 0.05 μl/s, starting at a drop volume of 20 μl. The measured advancing and receding contact angles for planar glass slides coated with Hydrobead Standard (a commercially available hydrophobic coating) were 169° ± 3° and 168° ± 4°, respectively. The measured advancing and receding contact angles for planar glass slides coated with Glaco (a commercially available superhydrophobic coating) were 170° ± 5° and 165° ± 3°, respectively. Contact angle for water droplet on Hydrobead coating is shown in fig. S2D, while contact angle hysteresis for all studied liquid systems is shown in fig. S2E.

### Sliding angles

Sliding angles were measured by depositing a 20-μl water droplet on a planar superhydrophobic surface fixed to a tilt platform. Then, the plate was inclined slowly until the droplet started to move. The whole process was imaged by a high-speed camera (Phantom v1610 with Canon macro photo lens MP-E 65 mm) at a frame rate of 1000 frames/s (fps). Sliding angles have been evaluated from the acquired image sequence using MATLAB. Measured sliding angles for all studied liquid systems are shown in fig. S2F.

### Viscosity measurements

Viscosities for the three different liquid systems used in this work (glycerol/water, PEO/water, and sucrose/water solutions) were measured by conventional 25-mm-diameter cone-plate rheology (Physica MCR 300, Anton Paar) at room temperature. A steady shear test was performed at shear rates between 1 and 1000 s^−1^. Viscosity values were calculated by averaging the viscosity between 10 and 100 s^−1^. The measured viscosities are given in table S1.

### Surface tension measurements

Surface tensions of the three different liquid systems used in this work (glycerol/water, PEO/water, and sucrose/water solutions) were measured with the pendant droplet method by using a conventional optical tensiometer (Attension Theta). Images of the pendant droplet were taken for 1 min at the rate of one image per second. The measured densities and surfaces tensions are given in table S1.

### Scanning electron microscopy imaging

Scanning electron microscopy (SEM) imaging was carried out with Zeiss Sigma VP. Samples used for SEM imaging were superhydrophobic capillaries coated with Hydrobead Standard and planar glass slides coated with Hydrobead Standard and Glaco. Capillaries were carefully cut to not destroy the coating inside the tube and placed on the carbon tape attached to an aluminum stub and coated with 2-nm gold coating using Leica EM ACE600 high-vacuum sputter coater before imaging. The images were taken at low acceleration voltage of 2.0 kV with an Everhart-Thornley detector (SE2 detector).

### Atomic force microscopy imaging

Atomic force microscopy (AFM) imaging was carried out with Veeco Dimension 5000 Scanning Probe Microscope equipped with NanoScope V controller (Veeco Inc., Santa Barbara, CA, USA) and HQ:NSC14/AlBS tips (with a nominal radius of 8 nm; MicroMasch). Samples used for AFM imaging were glass slides coated with Hydrobead Standard and Glaco Mirror Coat Zero.

### Droplet motion on inclined superhydrophobic surfaces

A 5-μl droplet was dispensed by a finnpipette on an inclined surface. The inclination angle was 30°. The droplet motion on the inclined superhydrophobic surface was imaged with a Phantom v1610 high-speed camera and Canon macro photo lens MP-E 65 mm at a frame rate of 1000 fps.

### Droplet motion inside open superhydrophobic capillaries

A 15-mm-long droplet was placed by pipette inside a 4.20–mm–inner diameter superhydrophobic capillary, taking care that the pipette did not touch the inside wall of the capillary. The inclination angle was 30°. Droplet motion was imaged by Phantom v1610 high-speed camera and Canon macro photo lens MP-E 65 mm at a frame rate of 1000 fps.

### Droplet motion inside closed superhydrophobic capillaries

The droplet was placed by a pipette inside a 4.20–mm–inner diameter superhydrophobic capillary, taking care that the pipette did not touch the inside wall of the capillary. The droplet motion inside the closed superhydrophobic capillary was imaged with a Canon 70D camera. Images were taken at the rate of one image per second using a Canon macro photo lens MP-E 65 mm. For more details, see the Supplementary Materials. An illustration of the setup is shown in fig. S1A.

### Modification of fluorescent particles

The tracer particles used in this work are fluorescent polyethylene microspheres (fluorescent Polyethylene Microspheres, Cospheric, 45- to 53-μm size). The particles were fluorescent green (density 1.002 g/cm^3^), fluorescent red (densities 1.091 and 1.20 g/cm^3^), and fluorescent blue (density 1.134 g/cm^3^). Particles were originally hydrophobic and not dispersible in the liquids. To modify them from hydrophobic to hydrophilic and make them dispersible in our studied liquids, a small amount of particle powder was placed inside a half-open vial for a 5-min oxygen plasma treatment. Afterward, the particles were dispersed in liquids of similar densities. To ensure the best possible density matching between particles and droplet fluid, the dispersion was centrifuged for 15 min. Sedimented and creamed particles were discarded, and only density-matched particles were collected, stored for 24 hours, and then used in experiments.

### Tracer particle experiments

For studying the internal flow of liquid droplets, density-matched tracer particles were used (see the fluorescent particle preparation described above). Fluorescent particles were used for better visualization of internal flow due to the semitransparent superhydrophobic coating. The particle motion inside the closed superhydrophobic capillaries were imaged under exposure of ultraviolet (UV) light (10PCS/lot 110V-240V T5 UV lamp 8W Black Light Blue) using a Canon 70D camera at the frame rate of 24 fps. A Canon macro photo lens MP-E 65 mm was used. See the Supplementary Materials for more details. An illustration of the setup is shown in fig. S4.

### Image analysis

The droplet motion was analyzed in MATLAB by tracking droplet position as a function of time [*x*(*t*)] (see fig. S1, C to F). The tracer particle motion was analyzed in MATLAB by tracking their positions and extracting their trajectories and velocities (see the Supplementary Materials for more details). The average internal flow velocity *U*_D_ was calculated as a weighted average, considering the cylindrical geometry. To avoid getting zero velocities, especially for low-viscosity liquids where symmetry is broken, the absolute values for the particle velocities were used in the calculations.

### Reflection interference contrast microscopy

In RICM measurements, the liquid droplet interface was raster scanned with focused beam of monochromatic light with wavelength of 458 nm. The reflected light was captured through the pinhole of a confocal scanner. This allows only for reflected light from the focal plane to reach the photomultiplier tube of the scanner for image formation. In the presence of a thin air film trapped between liquid droplet and capillary surface, the light reflected from the two interfaces interferes, resulting in a fringe pattern.

## Supplementary Material

aba5197_Movie_S5.mp4

aba5197_Movie_S2.mp4

aba5197_Movie_S3.mp4

aba5197_SM.pdf

aba5197_Movie_S4.mp4

aba5197_Movie_S1.mp4

## SUPPLEMENTARY MATERIALS

Supplementary material for this article is available at http://advances.sciencemag.org/cgi/content/full/6/42/eaba5197/DC1
