# Supplementary material for: Viscosity-enhanced droplet motion in sealed superhydrophobic capillaries
Source: Sci Adv. 2020 Oct 16;6(42):eaba5197. doi: 10.1126/sciadv.aba5197 (PMC7567596; doi:10.1126/sciadv.aba5197)
Supplement: aba5197_SM.pdf [file aba5197_SM.pdf]

Science Advances
AAAS

advances.sciencemag.org/cgi/content/full/6/42/eaba5197/DC1

Supplementary Materials for

Viscosity-enhanced droplet motion in sealed superhydrophobic capillaries

Maja Vuckovac, Matilda Backholm, Jaakko V. I. Timonen*, Robin H. A. Ras*

*Corresponding author. Email: robin.ras@aalto.fi (R.H.A.R.); jaakko.timonen@aalto.fi (J.V.I.T.)

Published 16 October 2020, *Sci. Adv.* **6**, eaba5197 (2020)
DOI: 10.1126/sciadv.aba5197

The PDF file includes:

Sections S1 to S11
Table S1
Figs. S1 to S8
Legends for movies S1 to S5

Other Supplementary Material for this manuscript includes the following:

(available at advances.sciencemag.org/cgi/content/full/6/42/eaba5197/DC1)

Movies S1 to S5

Supplementary Materials

S1. Properties of the liquid systems used in the experiments

The effect of viscosity and surface tension on the droplet motion was studied by using three different systems: water solutions of glycerol (Glycerol, Sigma Aldrich), sucrose (Sucrose, VWR) and polyethylene oxide polymer (PEO, Sigma Aldrich) with a molecular weight of 800 kg mol^{-1}. The studied systems were chosen in such a way that by increasing the concentration of glycerol, sucrose, or PEO in water solutions, the viscosity of these systems increases. On the other hand, by increasing the concentration of glycerol and PEO in water solution, surface tension for these systems decreases while it increases for sucrose solutions when the concentration of sucrose in water solution is increased.

Viscosities of the systems used in this work were measured by conventional 25 mm diameter cone-plate rheology (Physica MCR 300, Anton Paar) at room temperature (about 23 °C). A steady-shear test was performed at shear rates between $1-1000\ s^{-1}$. Viscosity values were calculated by averaging the viscosity between 10 and 100 s^{-1}. The measured viscosities are given in Table S1.

Surface tensions were measured with the pendant droplet method using approximately 5 µl liquid droplet in air by conventional optical tensiometer (Attension Theta). Densities were measured using Helmholtz syringe by measuring the mass of the known volume of the liquid and were calculated over mass and volumes, $\rho = m/V$, where ρ and m are the density and the mass of the liquid, respectively. The measured surface tensions and densities are given in Table S1.

The water solutions of glycerol and sucrose behave as Newtonian fluids, while for the polymer (PEO) with the high molecular weight we need to check how it follows Newton's law of viscosity. This was done by calculating overlap concentration ((*32*) from the main text) , c^*, which is the concentration at which the polymers start to overlap in the solution, and non-Newtonian (e.g. viscoelastic) fluid properties could be expected. For molecular weight close to molecular weight of the polymer used in this study (~800 kg mol^{-1}), overlap concentration is around c* ~ 0.2wt% and can be calculated using the formula(*1*): $c^* = 3\,M_w / (4\,\pi\,N_A\,R_g^3)$, where M_w is the molecular weight, N_A is the Avogadro number, and $R_g = 0.02\,M_w^{0.58}$ is the radius of gyration of the polymer (in nm). In our case, only the 0.1% PEO/water solution can be assumed to behave as Newtonian, whereas the 9% and 20% PEO/water solutions need to be considered as entangled polymer solutions. Hence, in scaling model from the main text, we are not

considering 9% and 20% PEO/water solutions, as the main model approximation is that fluids behave as Newtonian fluids. Even though PEO/water solutions behave as non-Newtonian fluids, it is interesting that the droplet velocities for these solutions coincide on the same curve with the Newtonian fluids when they are plotted versus viscosity (see Fig. 1 in the main text).

Table S1. Measured viscosities, surface tensions and densities for the three different systems used in this work.

Volume fraction	Viscosity (mPa s)	Surface tension (mN m^{-1})	Density (kg m^{-3})
Glycerol in water			
0 %	1.005 ± 0.097	72.7 ± 0.3	1000.5 ± 0.7
10 %	1.311 ± 0.074	72.1 ± 0.4	1022.1 ± 0.6
20 %	1.769 ± 0.059	71.6 ± 0.3	1046.9 ± 0.4
30 %	2.501 ± 0.063	70.5 ± 0.3	1072.7 ± 0.5
40 %	3.75 ± 0.25	69.4 ± 0.4	1099.3 ± 0.4
50 %	6.05 ± 0.55	68.2 ± 0.3	1126.3 ± 0.7
60%	10.96 ± 0.61	67.3 ± 0.4	1153.8 ± 0.8
70 %	22.94 ± 0.49	65.6 ± 0.3	1181.2 ± 0.6
80 %	62.00 ± 0.7	64.9 ± 0.2	1208.1 ± 0.9
90 %	234.6 ± 3.5	64.6 ± 0.2	1235.1 ± 0.5
95 %	545.0 ± 4.6	63.9 ± 0.3	1248.5 ± 0.5
98 %	974.0 ± 7.1	63.6 ± 0.3	1256.5 ± 0.8
100 %	1499.0 ± 10.5	63.2 ±0.3	1260.8 ± 0.9
Sucrose in water			
40 %	15.0 ± 0.3	73.0 ± 0.3	1178.5 ± 0.6
60 %	56.8 ± 0.8	75.1 ± 0.3	1288.7 ± 0.9
72 %	1019.9 ± 8.2	79.0 ± 0.5	1362.2 ± 1.1
PEO in water			
0.1 %	1.2 ± 0.1	62.1 ± 0.4	1070.2 ± 0.8
9 %	3.3 ± 0.4	44.7 ± 0.4	1150.0 ± 0.7
20 %	7.4 ± 0.7	28.1 ± 0.5	1210.1± 1.6

S2. Droplet motion inside a sealed superhydrophobic capillary

To study droplet motion inside superhydrophobic capillaries, we used NMR tubes with 4.20 mm inner diameter. The capillaries were cut to get approximately 15 cm long open-ended capillaries. Then they were cleaned in an alkaline solvent (Deconex 11 Universal, VWR) in ultrasonicated bath for 30 min, rinsed thoroughly with Milli-Q water and dried under nitrogen flow. After that, capillaries were coated with a commercially available coating solution (Hydrobead Standard) by placing one droplet of this solution into a capillary. Afterwards, they were dried carefully by nitrogen flow and annealed for 10 min at 110°C. The resulting capillaries are superhydrophobic and semi-transparent, which allows us to monitor droplet motion.

A schematic illustration of the experimental set-up is presented in Fig. S1A. We study in more detail droplet motion in a vertical superhydrophobic capillary closed from both ends. After placing the droplet inside the capillary, the relative pressure sensor (Dwyer, Series 475) was connected to both sides of the capillary using silicone tubing to measure pressure during the experiment. Pressure sensors were connected carefully, taking care not to have air leaking. The capillary was mounted vertically using holders, and the droplet motion was imaged from the side by the camera (Canon 70D with Canon macro photo lens MP-E 65 mm), taking one image per second.

The motion of a 15 mm long liquid droplet was studied for ten different capillaries with ten repetitions for each capillary. Used solutions were glycerol/water, PEO/water, and sucrose/water solutions (see Table S1). Experimentally measured pressures are plotted as a function of theoretically calculated hydrostatic pressure ($P = \rho g L$, where ρ is liquid density, g is the acceleration due to gravity, and L is the length of the droplet through the droplet axis from one to another end of the droplet) and shown in Fig. S1B. The linear relation between experimentally measured and theoretically calculated pressures suggests that hydrostatic pressure is driving the droplet downwards. All experiments were carried out at room temperature.

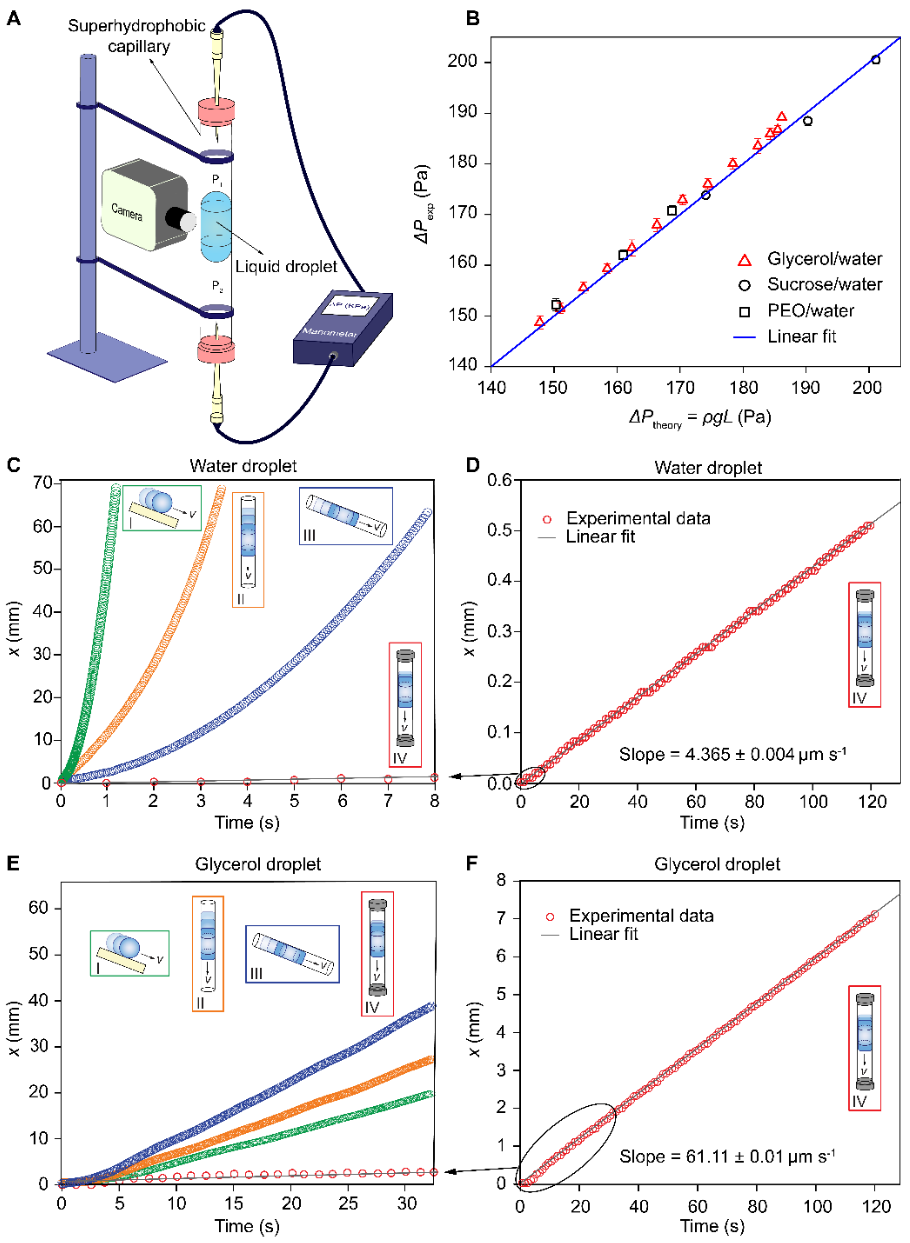

Fig. S1. Imaging droplet motion inside a sealed superhydrophobic capillary with tracked position of the droplet as a function of time for inclined superhydrophobic surfaces and inside superhydrophobic tubes. (**A**) A schematic illustration of the experimental set-up for imaging droplet motion inside the closed superhydrophobic capillary. The droplet motion is monitored from the side by the camera and pressures inside the tube were measured during the experiment. (**B**) Experimentally measured pressures as a function of theoretically calculated pressures for a 15 mm long droplet for all liquid systems used in work. The solid line is a linear fit with a slope of 1 that goes through the origin. (**C**) Position of the 5 μl water droplet for a superhydrophobic inclined plane, a 15 mm water droplet in vertical and inclined open-ended superhydrophobic capillary and in a closed superhydrophobic capillary as a function of time. (**D**) The full position of the water droplet (the first data point on Fig. 1 in the main text) as a function of time. (**E**) Position of the 5 μl glycerol droplet for a superhydrophobic inclined plane, a 15 mm glycerol droplet in a vertical open-ended and inclined open-ended superhydrophobic capillary, and in a closed superhydrophobic capillary as a function of time. (**F**) The full position of the glycerol droplet (the last data point on Fig. 1 in the main text) as a function of time. The inner diameter of the capillary was 4.20 mm. For (**C**) and (**E**), the inclination angle for both plane and open-ended capillary was 30°.

The experimental results were analyzed using Matlab, where the droplet position was monitored as a function of time. The raw data is presented in Fig. S1C-F. In Fig. S1C are shown tracking positions for a 5 µl water droplet moving on a plane inclined by 30° (I), a 15 mm water droplet moving inside an open-ended vertical (II) and open-ended inclined by 30° superhydrophobic capillary (III), and the first 8 s for a 15 mm water droplet moving inside the vertical closed superhydrophobic capillary (IV). The full tracking position for the 15 mm long water droplet is presented in Fig. S1D indicating that the water droplet is moving at a constant speed followed by small plateaus due to droplet pinning. The tracking positions for a 5 µl glycerol droplet moving on an inclined plane at 30° (I), a 15 mm glycerol droplet moving inside open-ended vertical (II) and open-ended inclined at 30° (III) superhydrophobic capillary, and the first 32 s for a 15 mm glycerol droplet moving inside a vertical closed superhydrophobic capillary (IV) are shown in Fig. S1E. The full tracking positions for the 15 mm long glycerol droplet (Fig. S1F) show that high-viscosity droplet needs a few seconds to find steady-state, after which it is moving at a constant speed along the tube.

S3. Characterisation of superhydrophobic coating

The characterisation of the superhydrophobic coating was done by imaging and measuring wetting properties of the coating. Imaging of superhydrophobic coating was performed using scanning electron microscopy (SEM) and atomic force microscopy (AFM). Characterisation of wetting properties of superhydrophobic capillaries and superhydrophobic surfaces was done by measuring contact angles, contact angle hysteresis and sliding angles.

Since the inner wall of a capillary tube is curved, it is difficult to characterize the coating inside the capillaries by above mentioned conventional techniques. Thus, planar surfaces coated similarly have been prepared and characterised, assuming that results are comparable. Microscopy glass slides were cleaned by ultrasonication in an alkaline solvent (Deconex 11 Universal, VWR), rinsed thoroughly with Milli-Q water and dried under nitrogen flow. Afterwards, commercial Hydrobead Standard was applied, and the glass slides were dried under ambient conditions and annealed for 10 min at 110 °C.

The SEM imaging was carried out with a Zeiss Sigma VP. Capillaries were cut along the longitudinal direction carefully not to destroy the coating inside the tube while planar surfaces were used as synthesised. Samples were sputtered with a thin layer of gold (2 nm)

using Leica EM ACE600 high vacuum sputter coater before imaging. The images were taken at a low acceleration voltage of 2.0 kV with Everhart-Thornley detector (SE2 detector). Supplementary Figure S2A and B show representative SEM images of Hydrobead Standard coating applied on the planar substrate and on the inner capillary wall, respectively. Images were scanned at the same magnification, and it can be noticed that on the same length scale, coating looks similar, especially for the zoom-in area on single hydrophobic bumps. Furthermore, SEM images of the superhydrophobic coating inside the capillary suggest that the plastron is supported by hydrophobic bumps that are randomly distributed with a spacing of a few tens of micrometers (Fig. S2A and B).

The surface roughness was obtained by AFM imaging with Veeco Dimension 5000 Scanning Probe Microscope equipped with Nanoscope V controller (Veeco, Inc., Santa Barbara, CA, USA) and HQ:NSC14/AlBS tips (with a nominal radius of 8 nm, Micro-masch). As mentioned before, the capillary inner surfaces could not be imaged by AFM due to the curvature. Since SEM imaging shows that the coating structure is similar in both planar and curved substrates, flat samples (glass slides coated with Hydrobead Standard) were used for the AFM imaging. A small area of 50 μm x 50 μm was scanned on five different spots close to each other, showing very similar AFM profiles. A representative 3D AFM reconstruction of a scanned area together with a 2D AFM profile along one line (for y = 20 μm) is shown in Fig. S2C. The average roughness is found to be $7.2 \pm 1.2\,\mu$m which gives an estimation of the plastron thickness (Fig. S2C).

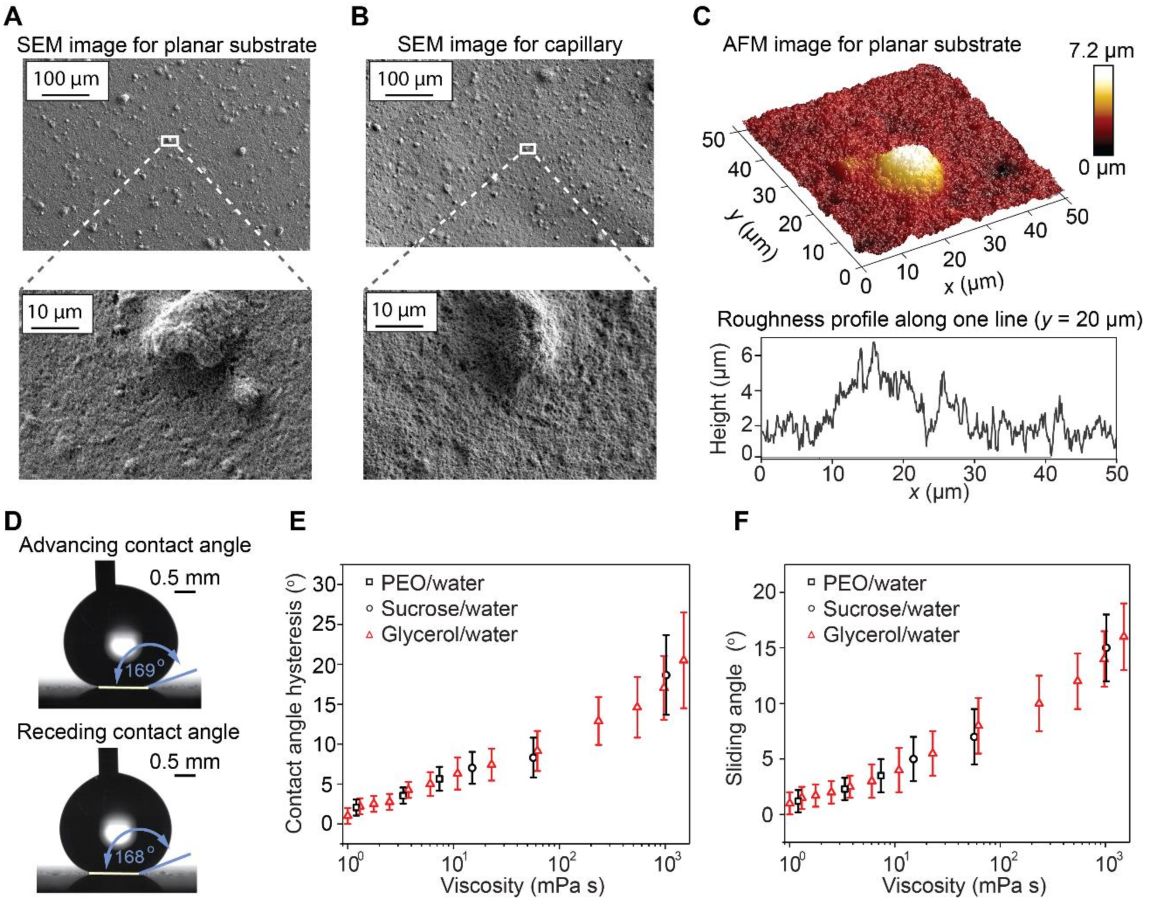

Fig. S2. Characterisation of the Hydrobead coating. (**A**) SEM images of the coating on the planar substrate. (**B**) SEM images of the inner surface of the capillary. (**C**) 3D AFM image and 2D AFM profile on a single hydrophobic bump. (**D**) Advancing contact angle and receding contact angle for water droplet on the planar substrate. (**E**) Contact angle hysteresis as a function of viscosity on the superhydrophobic surface. (**F**) Sliding angle as a function of viscosity on the surface. In both (**E**) and (**F**) data points from left to right correspond to PEO/water solutions with volume fraction of PEO 0.1%, 9% and 20%, for sucrose/water solutions with volume fraction of sucrose 40%, 60% and 72% and glycerol/water solutions with volume fraction of glycerol 0%, 10%, 20%, 30%, 40%, 50%, 60%, 70%, 80%, 90%, 95%, 98% and 100%. Each data point is the average of five measurements, while the error bars denote standard deviation.

Contact angles on the planar superhydrophobic surface were measured using the sessile droplet method by a conventional optical tensiometer (Attension Theta) with an automated liquid pumping system. Advancing contact angles were measured by placing a 2 μl droplet on the surface and increasing its volume to 20 μl, at a rate of 0.05 μl/s. Receding contact angles were measured by decreasing the droplet volume at a rate of 0.05 μl/s, starting from a droplet volume of 20 μl. The measured advancing contact angle, θ_A, and receding contact angle, θ_R, for water droplet on the planar substrate are 169° ± 3° and 168° ± 4°, respectively (Fig. S2D).

The reported contact angles are the average of five measurements on different locations of the substrate while the errors denote standard deviation.

Contact angle hysteresis and sliding angles on planar superhydrophobic surfaces for all solutions used in this work were measured and are shown in Fig. S2E-F. The contact angle hysteresis calculated as a difference between advancing, θ_{A}, and receding, θ_{R}, contact angle, $\Delta\theta = \theta_{\mathrm{A}} - \theta_{\mathrm{R}}$, is very small ~1° for water droplet and increases up to ~20° for glycerol droplet (Fig. S2E).

Sliding angles were measured by depositing a 20 µl water droplet on a planar superhydrophobic surface fixed to a tilt platform. Then the plate was inclined slowly until the droplet started to move. The whole process was imaged by a high-speed camera (Phantom V1610 with Canon macro photo lens MP-E 65 mm) at a frame rate of 1000 fps. Sliding angles have been evaluated from the acquired image sequence with house-written Matlab script, and it shows to be ~1° for the water droplet and increase up to 15° for the glycerol droplet (Fig. S2F).

S4. The influence of the plastron existence on the droplet motion

Topographically rough surfaces can support a metastable air layer, also known as a plastron, between the solid and liquid. This is the well-known Cassie-Baxter state ((*4*, *8*) from the main text), and liquid droplets rest partly on air trapped within the nano- and microstructures of the rough surface. The absence of the plastron is characteristic for the Wenzel state ((*8*, *28*) from the main text) and hydrophobic surfaces ((*8*) from the main text), where liquid fills the space between the roughness of the surface.

To test how the existence of the plastron affects the droplet motion, we prepared hydrophobic capillaries and capillaries with droplet in the Wenzel state. The 4.20 mm inner diameter NMR capillaries were cut to get open-ended capillaries and were cleaned by ultrasonication in an alkaline solvent (Deconex 11 Universal, VWR) for 30 min. Then, we rinsed them thoroughly with Milli-Q water and dried under nitrogen flow. Afterwards, the capillaries were immersed in 0.002 M octadecylphosphonic acid / tetrahydrofuran solution (ODPA/THF) for 35 minutes, followed with annealing for 48 h at 120 °C. The resulting capillary is hydrophobic and transparent (Fig. S3A). The ODPA/THF solution was prepared by

dissolving octadecylphosphonic acid powder (ODPA, Sigma Aldrich) into tetrahydrofuran (THF, Sigma Aldrich) by ultrasonication for utterly dissolving the ODPA powder.

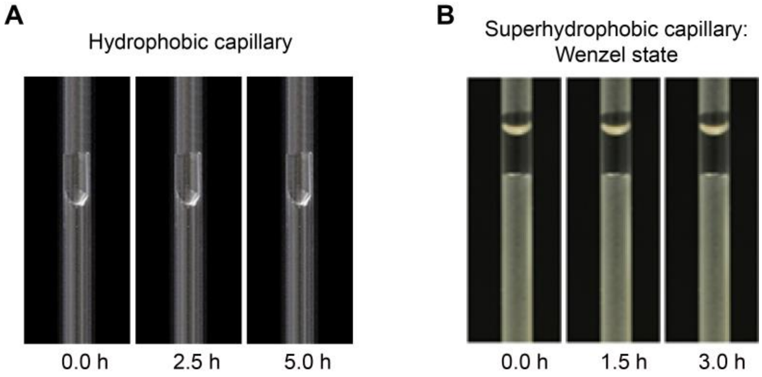

Fig. S3. Droplets inside capillaries without air layer (plastron) trapped between solid and liquid. (**A**), Water droplet in 4.20 mm inner diameter capillary with a smooth hydrophobic coating (ODPA/THF, described in Methods), and (**B**) 50% ethanol/water droplet in the Wenzel state in 4.20 mm inner diameter superhydrophobic capillary.

The Wenzel state was obtained by using superhydrophobic capillaries coated with Hydrobead (described in Section S1) and a droplet of 50/50 water/ethanol solution. In this case, as a final result, the droplet wets the wall of the coated capillary (Fig. S3B). In both cases, the hydrophobic capillary and the capillary in the Wenzel state, the plastron is not present, as can be noticed by the shape of the droplet. The droplet remains stationary in the capillary (Fig. S3) for both cases.

S5. Imaging the flow field inside the droplet

For studying the internal flow of liquid droplets, we used tracer particles (fluorescent polyethylene Microspheres, Cospheric) of 45-53 µm size. The used microspheres were fluorescent green (density 1.002 g/cm^3), fluorescent red (densities 1.091 g/cm^3 and 1.20 g/cm^3) and fluorescent blue (density 1.134 g/cm^3). Particles are initially hydrophobic and do not disperse in the liquids. To modify them from hydrophobic to hydrophilic, and make them dispersible in studied liquids, a small amount of particle powder was placed inside a half-open vial for 1 min O_2 plasma treatment (Diener electronics). Then the vial was shaken and a second plasma treatment carried out for 1 min. This was repeated 5 times, in total 5 min plasma treatment. Afterward, particles were dispersed in liquids, centrifuged for 15 min (max RCF 200 x g). Particles that sedimented or creamed were discarded and density matched particles were

collected. The density matched particles were then stored for one day to ensure the best possible density matching, and those were used in experiments.

An illustration of the experimental set-up for imaging the flow inside the liquid droplet is shown in Fig. S4. The droplet with tracer particles is placed in a carefully sealed superhydrophobic capillary to avoid leaking. Then, the capillary is immersed in a glass container with flat walls filled with the same liquid as used in the drop inside the capillary. In this way, we match refractive indices and eliminate curved edges due to the tubular geometry of the capillary. Before we start imaging the flow, we wait 30 min to stabilize the flow inside the droplet. Then, the particle motions were imaged from the side by the camera (Canon 70D with Canon macro photo lens MP-E 65 mm at a frame rate of 24 fps). The longpass filters (GG 495 and OG 570, SCHOTT) were used to remove excitation light and increase contrast. When UV light illuminates the droplet with tracer particles, long wavelength light (green and red) can pass through these filters while UV wavelengths are blocked. In other words, we used GG 495 filters for imaging green tracer particles and the OG 570 for imaging red tracer particles. The videos were analyzed using a home-written particle image velocimetry (PIV) Matlab script. The particles' position in x and y directions over time was monitored, and their velocity vectors were extracted, as shown in Fig. 2A and B in the main text and the average velocity of all particles was calculated (average internal flow velocity from Fig. 2C in the main text).

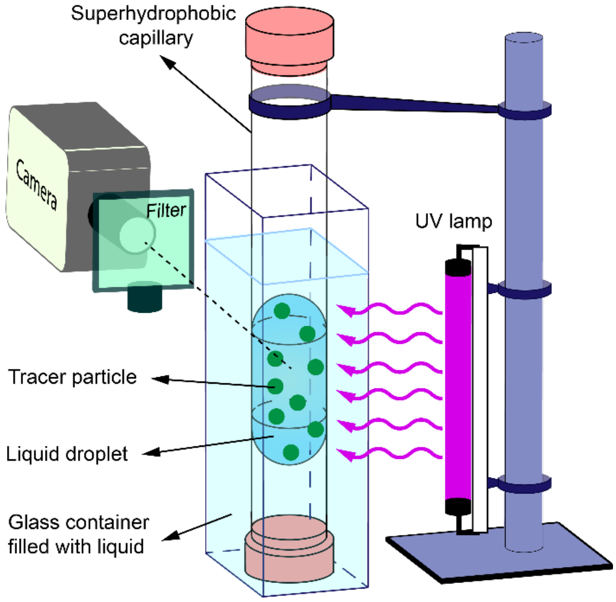

Fig. S4. A schematic illustration of the experimental set-up for imaging motion of tracer particles in a liquid droplet. The superhydrophobic capillary, together with liquid droplet and tracer particles, is immersed in a glass container filled with liquid. The droplet is illuminated by a UV lamp, and the tracer

particles motion is monitored from the sides with a high-speed camera. The filters remove the excitation light, letting the fluorescence light to pass to the camera.

S6. The influence of the plastron existence on the flow field inside the droplet

To verify that the internal flow in the liquid droplet is determined by the presence of the plastron, we also did tracer particle experiments where a plastron does not exist. In these experiments, we used a 45:50:5 ethanol:water:glycerol solution. As shown in Fig. S5, the tracer particles are not moving in the absence of the plastron.

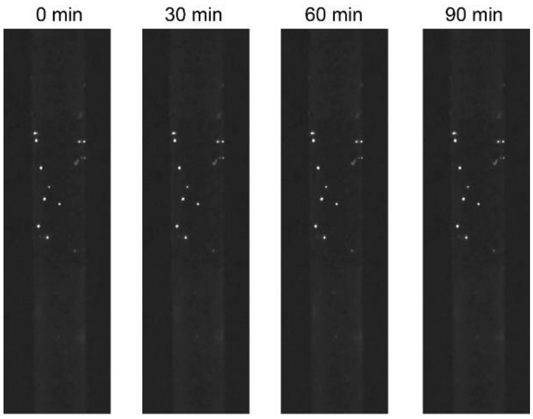

Fig. S5. The flow field in the liquid droplet inside capillary without air layer (plastron) trapped between solid and liquid. The droplet of a 45:50:5 ethanol:water:glycerol solution in the Wenzel state in 4.20 mm inner diameter superhydrophobic capillary.

S7. Dissipation mechanism of the system

To model the droplet velocity v_{D}, we need to carefully validate the dissipation mechanisms of our system. Dissipation occurs in the droplet and air film through viscous friction forces: $F_{D,\eta} \sim \eta_{\mathrm{D}} L(U_{\mathrm{D}} - v_{\mathrm{D}})$ and $F_{\mathrm{A},\eta} \sim \frac{\eta_{\mathrm{A}} R L U_{\mathrm{A}}}{\delta} \sim \frac{\eta_{\mathrm{A}} L v_{\mathrm{D}} R^2}{\delta^2}$, as well as inertial friction forces: $F_{\mathrm{D},\rho} \sim \rho R L (U_{\mathrm{D}} - v_{\mathrm{D}})^2$ and $F_{\mathrm{A},\rho} \sim \rho_{\mathrm{A}} R L U_{\mathrm{A}}^2 \sim \frac{\rho_{\mathrm{A}} R^3 L v_{\mathrm{D}}^2}{\delta^2}$. Fig. S6 shows both viscous and inertial dissipative forces for the droplet and air film and we can see that the viscous dissipation in the air film dominates in our system. The dissipative forces were calculated using the actual plastron thickness δ_{η}. The results remain almost unchanged when the dissipation forces are calculated using a fixed average plastron thickness δ, or the actual plastron thickness δ_{η} (Fig. 4C in the main text).

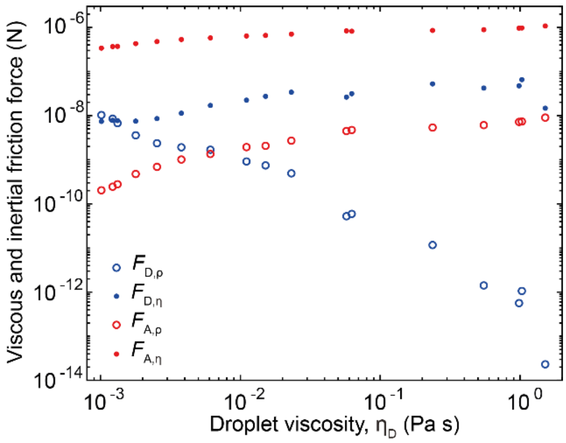

Fig. S6. Dissipation forces for droplets in closed superhydrophobic capillaries. Viscous and inertial friction forces calculated for the droplet and the air film using the actual plastron thickness δ_η (see main text for more details).

S8. Effect of droplet size and capillary radius on droplet velocity

As discussed in the main text, we modelled the droplet velocity v_D from the balance of viscous friction forces in air and driving gravitational force. Since both of these forces are linearly dependent of the droplet length L, the droplet velocity v_D should be independent of the droplet length. We performed experiments for different droplet lengths for water droplet and glycerol and found that droplet velocity does not depend on droplet length for long enough droplets, as shown in Fig. S8A. Droplets are considered to be long enough if they satisfy condition $l > 2R$, where l is the contact length, and $2R$ is the diameter of the capillary (Fig. S7A). For short droplets, the critical droplet length should be $L_\mathrm{C} > 2R$ in order to ensure trapping of the droplets inside the capillary (to have enough contact length l). Thus, small droplets have higher velocities due to spherical shape, small contact length l, and therefore smaller friction forces.

On the other hand, the droplet velocity v_D should depend on the capillary radius, which defines the flow rate of both liquid and air. We thus performed experiments with different capillary radii for ~180 µl water and glycerol droplets. As shown in Fig. S7B, the velocity of both the water and glycerol droplet increases with increasing capillary radius. This is in disagreement with Eq. (2) (main text). However, from Eq. (4) (main text), we can see that the interface deformation ε will increase with increasing capillary radius, resulting in a larger plastron thickness δ_η. Since we show that the droplet velocity is very sensitive to the plastron thickness δ_η (so that a larger plastron will speed up the droplets), this can explain the faster droplets for a larger capillary radius. In our system, droplet length L and capillary radius R are not independent values, and for the same droplet volume, droplet length increases when capillary radius decreases.

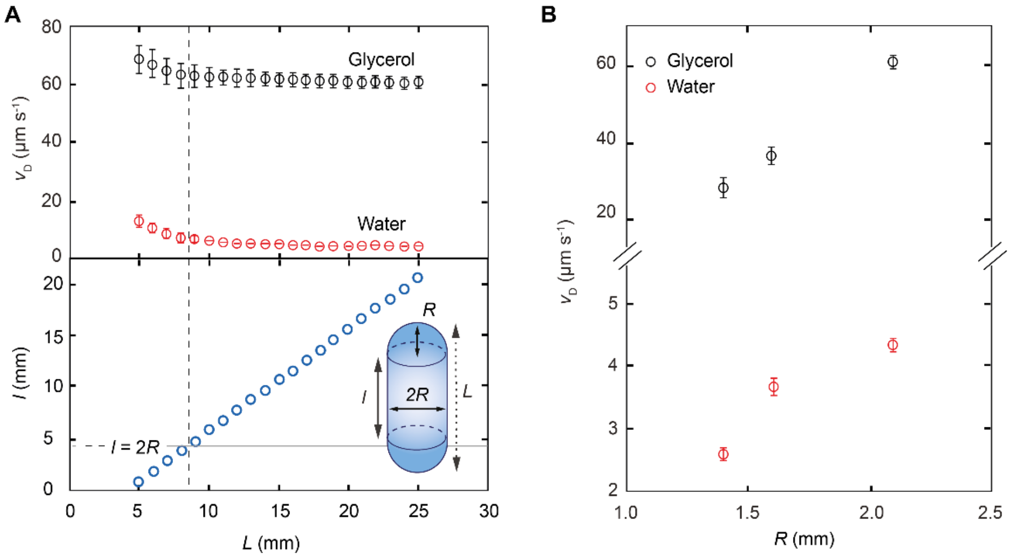

Fig. S7. Dependence of the droplet velocity on droplet length and capillary radius. (**A**) Droplet velocity v_D as a function of droplet length L (top), with assumed squeezed droplet shape, where R is the radius of the capillary, and l is the droplet contact length (= droplet length L minus hemispherical ends). Droplet contact length versus droplet length (bottom). The results are for the capillary with inner diameter $2R = 4.2$ mm. (**B**) Droplet velocity v_D as a function of the capillary radius R (1.4 mm, 1.6 mm and 2.1 mm from left to right), for ~180 µl water and glycerol droplets. Each data point in (**A**) top and (**B**) are averages of five measurements while the error bars denote standard deviations.

S9. Visualization of the droplet interface deformation

Imaging of the interface deformation for water and glycerol droplet was done using confocal reflection interference contrast microscopy (RICM) in vertical, closed superhydrophobic capillaries with an upright Zeiss LSM710 confocal and objective lens (5x) rotated 90° to horizontal orientation with a mirror. This allows us to image the dynamics of the liquid-air interface for moving droplets through the tube (Supplementary Movies S4 and S5). To partially eliminate the effect of curvature due to cylindrical geometry on the imaging, we glued a flat cover glass to the capillary with polydimethylsiloxane (PDMS), through which we imaged the plastron (thus schematic illustration in Fig. 6 is simplified). Quantitative determination of the thickness of the plastron could be possible using two-wavelength confocal RICM (*(33)* from the main text), but was found to be challenging in the current system due to difficulty of alignment of two wavelengths due to additional optics required to rotate the optical axis and the cylindrical geometry of the sample.

S10. The influence of surface roughness on droplet motion

To study droplet motion in superhydrophobic capillaries with different coatings, a transparent coating with a variety of well-controlled regular geometrical patterned surface structures would have been ideal. We found, however, that the synthesis of such a coating inside a capillary of small inner diameter (< 5 mm) and long length (approximately 15 cm) was extremely challenging. Despite these challenges to coat the inside wall of a capillary with superhydrophobic coating for more than 3 cm, we managed to synthesize a long superhydrophobic capillary with a different coating using another commercially available coating solution (Glaco Mirror Coat Zero, SOFT99). We used the same NMR tube as one described before. They were cleaned by ultrasonication in an alkaline solvent (Deconex 11 Universal, VWR) for 30 min, rinsed thoroughly by Milli-Q water and dried under nitrogen flow. Capillaries were coated with Glaco by filling the capillary with a solution using the pipette and left, filled with solution, for 1 h. Afterwards, the solution was removed, and capillaries were dried carefully at ambient conditions and annealed for 10 min at 110°C. The resulting capillaries are superhydrophobic and transparent. The coating characterisation was done by imaging with SEM and AFM, and by measuring contact angles. The results are shown in Fig. S8.

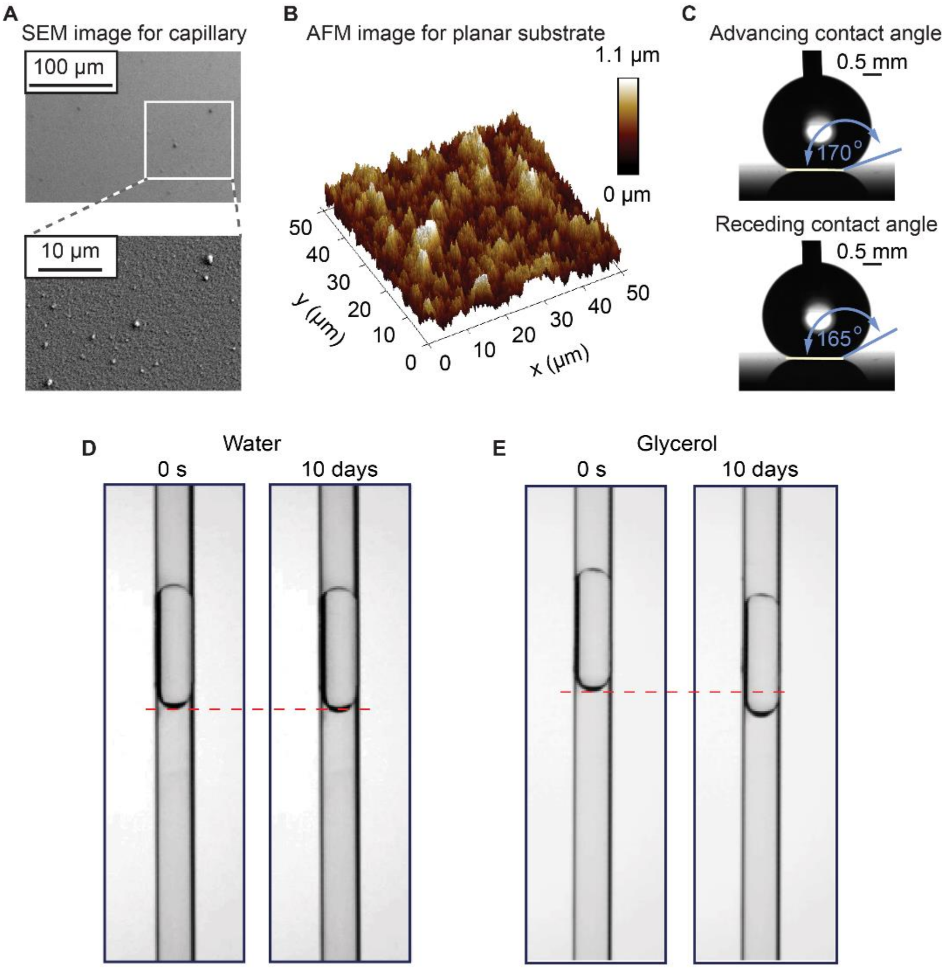

Fig. S8. Viscosity-enhanced droplet motion for the Glaco superhydrophobic coating. (**A**) SEM images of the inner surface of capillary and (**B**) 3D AFM image of a planar substrate coated with superhydrophobic coating Glaco. (**C**) Advancing contact angle and receding contact angle for water droplet on a planar substrate coated with Glaco Mirror Coat Zero. (**D**) Water droplet in its initial and final position showing that the droplet moved down 1.5 mm during 10 days. (**E**) Glycerol droplet in its initial and final position showing that droplet moved down 4 mm during 10 days. The capillary used for experiments shown in (**D**) and (**E**) is 4.2 mm inner diameter capillary.

Glaco Mirror Coat Zero solution has less rough topography (Fig. S8A and B, average roughness of 1.1 ± 0.2 μm) than Hydrobead shown in Fig. S2A-C. We obtained for Glaco-coated capillary that by decreasing the coating roughness, the time which is needed for the droplet to pass a millimeter is increased from minutes to days (Fig. S8D). Using this coating, we also noticed the same anomalous droplet motion: higher viscous glycerol droplet is moving faster than less viscous water droplet (Fig. S8E). These experiments, together with experiments performed with Hydrobead coating, indicate that viscosity-enhanced drop motion is general for closed superhydrophobic capillaries.

S11. Supplementary Movies

Movie S1. Motion of tracer particles inside the low-viscosity droplet (30% glycerol/water, η_D=2.5 mPa s).

Movie S2. Motion of tracer particles inside the intermediate-viscosity droplet (70% glycerol/water, η_D= 22.94 mPa s).

Movie S3. Motion of tracer particles inside the high-viscosity droplet (100% glycerol/water, η_D=1499 mPa s).

Movie S4. Interface deformation for low-viscosity droplet (0% glycerol/water, η_D=1 mPa s).

Movie S5. Interface deformation for high-viscosity droplet (100% glycerol/water, η_D=1499 mPa s).
